# Spinal Cord Involvement in Pediatric-Onset Metabolic Disorders With Mendelian and Mitochondrial Inheritance

**DOI:** 10.3389/fped.2020.599861

**Published:** 2021-01-14

**Authors:** Brahim Tabarki, Wejdan Hakami, Nader Alkhuraish, Kalthoum Tlili-Graies, Majid Alfadhel

**Affiliations:** ^1^Division of Pediatric Neurology, Department of Pediatrics, Prince Sultan Military Medical City, Riyadh, Saudi Arabia; ^2^Division of Neuroradiology, Department of Radiology, Prince Sultan Military Medical City, Riyadh, Saudi Arabia; ^3^Medical Genomics Research Department, King Abdullah International Medical Research Center, King Saud Bin Abdulaziz University for Health Sciences, King Abdulaziz Medical City, Ministry of National Guard-Health Affairs, Riyadh, Saudi Arabia; ^4^Genetics and Precision Medicine Department, King Abdulaziz Medical City, Ministry of National Guard Health Affairs, King Abdullah Specialist Children's Hospital, King Saud Bin Abdulaziz University for Health Sciences, Riyadh, Saudi Arabia

**Keywords:** spinal cord, Mendelian disease, mitochondrial disease, MRI, children

## Abstract

Previous reviews have described the features of brain involvement in pediatric-onset metabolic disorders with Mendelian and mitochondrial inheritance, but only a few have focused on spinal cord abnormalities. An increasing number of metabolic disorders with Mendelian and mitochondrial inheritance in children with predominant spinal cord involvement has been recognized. Spinal cord involvement may be isolated or may occur more frequently with brain involvement. Timely diagnosis and occasional genetic counseling are needed for timely therapy. Therefore, clinicians must be aware of the clinical, laboratory, and radiographic features of these disorders. In this review, we describe pediatric-onset metabolic disorders with Mendelian and mitochondrial inheritance and predominant spinal cord involvement. Furthermore, we provide an overview of these conditions, including background information and examples that require rapid identification, focusing on treatable conditions; that would be catastrophic if they are not recognized.

## Introduction

Among the most-affected systems in metabolic disorders with Mendelian and mitochondrial inheritance, the central nervous system, including the brain, is the most involved (1, 2). Owing to advances in neuroimaging and molecular genetic and/or biochemical testing, an increasing number of genetic and metabolic diseases of childhood with predominant spinal cord involvement is being recognized. Spinal cord involvement in Mendelian and mitochondrial diseases most frequently occurs with brain involvement. However, it might also be the disease manifestation as well (3–8). Prompt recognition of metabolic disorders with Mendelian and mitochondrial inheritance as a potential cause for a phenotype involving the spinal cord is critical for accurate patient management, as some of these diseases are potentially treatable and evoke broader genetic counseling implications for the affected families. In this review, we discuss common pediatric-onset metabolic disorders with Mendelian and mitochondrial inheritance and associated spinal cord abnormalities.

## Methods

Written informed consent was obtained from each minor's legal guardian for the publication of any potentially identifiable images or data included in this article.

The online database MEDLINE was used to perform a literature search for papers published between January 1985 and December 2019 without any date or language restrictions. We used a combination of relevant search terms, such as “children AND leukodystrophy AND spinal cord” (83 papers), “children AND leukoencephalopathy AND spinal cord” (385 papers), “children AND mitochondrial diseases AND spinal cord” (122 papers), “inherited metabolic disorders AND spinal cord AND children” (741 papers), and “vitamin AND spinal cord AND children” (126 papers). We independently reviewed articles to identify metabolic disorders with Mendelian and mitochondrial inheritance in pediatric patients with spinal cord involvement and systematically screened titles, abstracts, and full texts of the collected articles. Reviews and editorials were excluded (238 papers).

Pediatric-onset metabolic disorders with Mendelian and mitochondrial inheritance and spinal cord involvement were etiologically classified into three groups: (1) leukodystrophies, (2) mitochondrial diseases, and (3) deficiency-related metabolic diseases. The common pediatric-onset metabolic disorders with Mendelian and mitochondrial inheritance and spinal cord involvement are outlined in Tables 1, 2 (4–42).

**Table 1:** Common pediatric-onset metabolic disorders with Mendelian and mitochondrial inheritance with predominant spinal cord involvement.

**Classification**	**Disease**	**Genetic cause**	**Spinal cord involvement**	**Treatment**
Leukodystrophies	Adrenoleukodystrophy	*ABCD1*	Spinal cord atrophy Less common: involvement of corticospinal and corticopontine tracts	HSCT: can halt the cerebral demyelination if done early before neurological symptoms
	LBLS	*DARS2*	Involvement of the dorsal columns and lateral corticospinal tract of the spinal cord	Supportive care
	HBSL	*DARS*	Extensive spinal cord involvement	Supportive care
	Alexander disease	*GFAP*	Atrophy and signal intensity changes at the cervicomedullary junction	Supportive care
	Krabbe disease	*GALC*	Diffuse gadolinium enhancement of the lumbosacral nerve roots and cauda equina	Supportive care
	Metachromatic leukodystrophy	*ASA*	Diffuse gadolinium enhancement of the lumbosacral nerve roots and cauda equina	HSCT: can halt the cerebral demyelination if done early before neurological symptoms
	Spinal cerebrotendinous xanthomatosis	*CYP27A1*	Longitudinally extensive posterior and lateral column white matter abnormalities	Chenodesoxycholic acid 750 mg/day (oral)
	*RARS*-related disease	*RARS*	Upper spinal cord	Supportive care
	*EPRS*-related disease	*EPRS*	Posterior columns of the spinal cord	Supportive care
	*AARS2*-related disease	*AARS2*	Corticospinal tracts	Supportive care
	Pelizaeus–Merzbacher-like disease	*GJC2*	Involvement of the cervical spinal cord	Supportive care
	Aicardi-Goutieres syndrome	*ADAR*	Spinal cord swelling and hyperintensity of the central cord matter	Supportive care
Mitochondrial diseases	Leigh syndrome	>75 (monogenic)	Gray/white matter necrosis or demyelination	Supportive care
	Multiple mitochondrial dysfunctions syndrome 4	*ISCA2*	Extensive longitudinal involvement of spinal cord in particular of lateral corticospinal tract and dorsal column	Supportive care
	MELAS	*MTTL1 (>80%)* *Other mtDNA*	Gliosis in anterior and posterior horns and degeneration of corticospinal tracts and the posterior or lateral columns	L-arginine 500 mg/kg (IV), the 150–300 mg/kg/day oral L-carnitine L-citrulline
	MERRF	*MT-TK*	Neuronal loss in the anterior and posterior horns and severe neuronal loss in Clarke's column	Supportive care
	Kearns–Sayre syndrome	*mtDNA deletion*	Corticospinal tracts	Supportive care
	Infantile-onset spinocerebellar ataxia	*C10orf2*	Atrophy, mainly the cervical cord	Supportive care
	Mitochondrial recessive ataxia syndrome	*POLG1*	Atrophy, mainly posterior columns of spinal cord	Supportive care
	Pontocerebellar hypoplasia	*SLC25A46*	Loss of motor neurons in the anterior horns and “ballooning” appearance	Supportive care
	Chronic progressive external ophthalmoplegia	*C10orf2*	Involvement of the posterior columns	Supportive care
	KARS-related disease	*KARS*	“Track-like” calcifications along the whole spinal cord	Supportive care
	Leber's hereditary optic neuropathy	*ND6*	Demyelinating lesions of the spinal cord	Supportive care
	NDUFS6-related	*NUFS6*	Lateral cortico-spinal tract at the cervical region (Figure 2)	Supportive care
Vitamins	Biotinidase deficiency	*BTD*	Diffuse involvement of the spinal cord+_enlargement	Biotine 10–20 mg/day (oral)
	Biotin-Thiamine responsive basal ganglia disease	*SLC19A3*	Cervical spinal cord swelling (Figure 3)	Thiamine up to 1,500 mg/day Biotine 5 mg/kg/day
	Cobalamin-related remethylation disorders cblC, cblD, cblE, cblF, cblG, cblJ and MTHFR deficiency	*MTHFR, MMACHC (*Cbl C*), MTR (*Cbl G*), MTRR (*Cbl E*), LMBRD1 (*Cbl F*), C2Orf25* (Cbl D).	Symmetrical posterior spinal cord involvement	- Betaine 6–9 g/day (oral) - Hydroxy-Cobalamin 1 mg/day (IM) as initial treatment, monthly afterward. - Folate 5–10 mg/day (oral)
	Ataxia with vitamin E deficiency	*TTPA*	Symmetrical involvement of the posterior columns, particularly in the lower cervical segments	Vitamin E: 40 mg/kg (oral)
	Cerebral folate deficiency	*FOLR1*	Involvement of the posterior and lateral spinal cord	Folinic acid: 2–5 mg/kg/day
Miscellaneous	Friedreich's ataxia	*FXN*	Cervical atrophy	
	Wilson disease	*ATP7B*	Involvement of the cervical spinal cord, mainly posteriorly	Chelating agents (Penicillamine, Trientine, zinc acetate)
	HHH syndrome	*SLC25A15*	Spinal cord atrophy	- Low protein diet - Supplementation: arginine, citrulline, ornithine - Ammonia scavengers
	Arginase deficiency	*ARG1*	Atrophy with paucity of fibers in the cortical spinal tract bundle	- dietary protein restriction - pharmacologic ammonia scavengers
	Nonketotic hyperglycinemia	*GLRX5*	Central lesions of the upper spinal cord	Supportive care

*HSCT, Allogeneic hematopoietic stem cell transplantation; LBLS, leukoencephalopathy with brainstem, spinal cord involvement, and lactate elevation; HBSL, hypomyelination with brainstem and spinal involvement and leg spasticity; MELAS, Myoclonic epilepsy with lactic acidosis and stroke-like episodes; MERRF, myoclonic epilepsy with ragged red fibers*.

**Table 2:** Common treatable causes of metabolic myelopathy.

**Condition**	**Biochemical findings**	**Genetic cause**	**Treatment**
Biotinidase deficiency	- OAC (urine), - lactic acid (CSF), - biotinidase enzymatic activity	*BTD*	Biotine 10–20 mg/day (oral)
Biotine-Thiamine responsive basal ganglia disease	OAC (urine) Lactic acid (CSF)	*SLC19A3*	Thiamine up to 1,500 mg/day Biotine 5 mg/kg/day
Cobalamin-related remethylation disorders cblC, cblD, cblE, cblF, cblG, cblJ and MTHFR deficiency	Homocysteine, methionine, B12, folate (plasma); MMA (plasma or urine).	MTHFR, MMACHC (Cbl C), MTR (Cbl G), MTRR (Cbl E), LMBRD1 (Cbl F), C2Orf25 (Cbl D).	- Betaine 6–9 g/day (oral) - Hydroxy-Cobalamin 1 mg/day (IM) as initial treatment, monthly afterward. - Folate 5–10 mg/day (oral)
HHH syndrome	- Hyperornithinaemia - Hyperammonaemia - Homocitrullinuria	*SLC25A15*	- Low-protein diet - Supplementation: arginine, citrulline, ornithine - Ammonia scavengers
Arginase deficiency	- Plasma ammonia - Urinary orotic acid	*ARG1*	- dietary protein restriction - pharmacologic ammonia scavengers
Ataxia with vitamin E deficiency	Very low plasma vitamin E (α-tocopherol)	*TTPA*	Vitamin E: 40 mg/kg (oral)
Cerebral folate deficiency	- low levels of 5-methyltetrahydrofolate in the CSF - normal folate levels in the plasma	*FOLR1*	Folinic acid: 2–5 mg/kg/day
Wilson disease		*ATP7B*	Chelating agents (Penicillamine, Trientine, zinc acetate)

*OAC, organic acid chromatography; MMA, methylmalonic acid*.

## Leukodystrophies

Leukodystrophies are a heterogeneous group of heritable disorders that primarily affect the white matter of the central nervous system (8). They may affect both the brain and spinal cord (Table 1) (8–20). Their clinical manifestations often include ataxia, spasticity, and cognitive decline of variable severity.

### Adrenoleukodystrophy

X-linked adrenoleukodystrophy (X-ALD) is the most common peroxisomal disorder. It is caused by mutations in ABCD1, which encodes the peroxisomal membrane protein ALDP. The clinical spectrum of X-ALD ranges from isolated Addison's disease and slowly progressive myelopathy to brain involvement. Most patients with ALD develop adrenomyeloneuropathy in adulthood. Neuropathological studies have shown that distal non-inflammatory axonopathy mainly affects the corticospinal tracts and dorsal columns (8). Spinal cord imaging in ALD mainly reveals atrophy; however, other anomalies can be observed, including the involvement of the corticospinal and corticopontine tracts. Van de Stadt et al. showed that the cervical spinal cord in patients with ALD is smaller and flattened compared to that in controls, possibly due to atrophy of the dorsal columns, and can be a marker of disease severity (9). The treatment of X-ALD remains mainly supportive. Early hematopoietic stem-cell transplantation is a treatment option with variable results. However, treatment using Lorenzo's oil has not consistently resulted in a substantial improvement in affected individuals. No treatment is currently available for progressive myelopathy in X-ALD (8).

### Leukoencephalopathy With Brainstem, Spinal Cord Involvement, and Lactate Elevation

**Figure 1:**
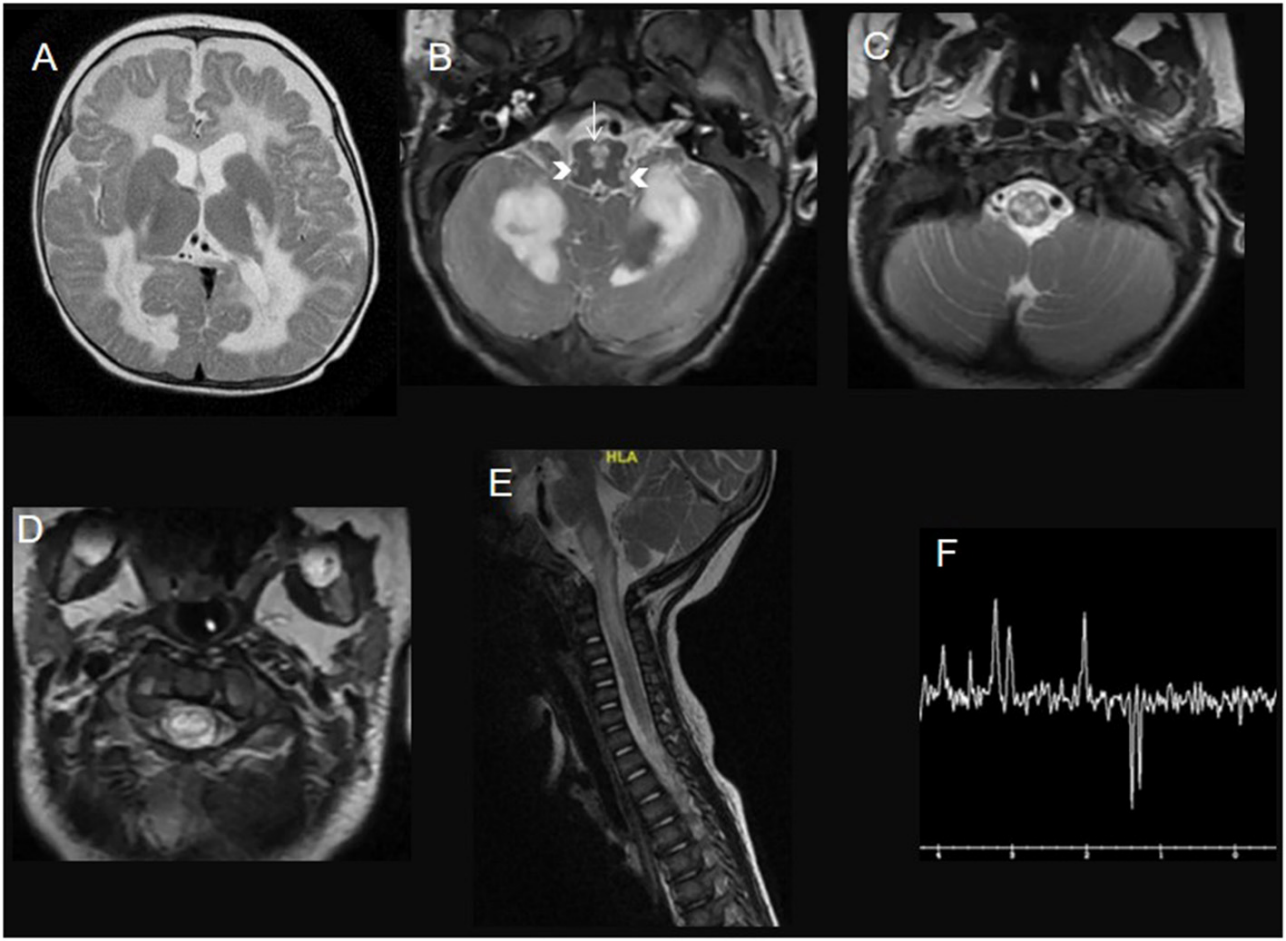
A 3-year-old child with leukoencephalopathy with the brainstem, spinal cord involvement, and lactate elevation due to *DARS2* mutation. Brain **(A–C)** and spinal cord **(D,E)** MRI T2WI with Long Echotime MRS **(F)** hyperintensity of the deep white matter tracts in the brain (involving the posterior limb of internal capsules and sparing the subcortical U fibers), the deep cerebellar white matter, the pyramids in the medulla (white arrow) and the inferior cerebellar peduncles (head arrows), the dorsal column and lateral corticospinal tracts over the rostral spinal cord. Long echo time MRS **(F)** shows an inverted prominent lactate peak at 144 ms.

This is an autosomal recessive disease caused by a homozygous or compound heterozygous mutation in the *DARS2* gene, which encodes a mitochondrial aspartyl-tRNA synthetase. Its clinical manifestations include progressive pyramidal, cerebellar, and dorsal column dysfunction, variable cognitive deficit or epilepsy. The MRI pattern is characteristic of abnormalities in the cerebral white matter, selective involvement of the brainstem and spinal cord tracts, and lactate elevation. Imaging of the spinal cord shows the involvement of the dorsal columns and lateral corticospinal tracts (Figure 1) (10).

**Figure 2:**
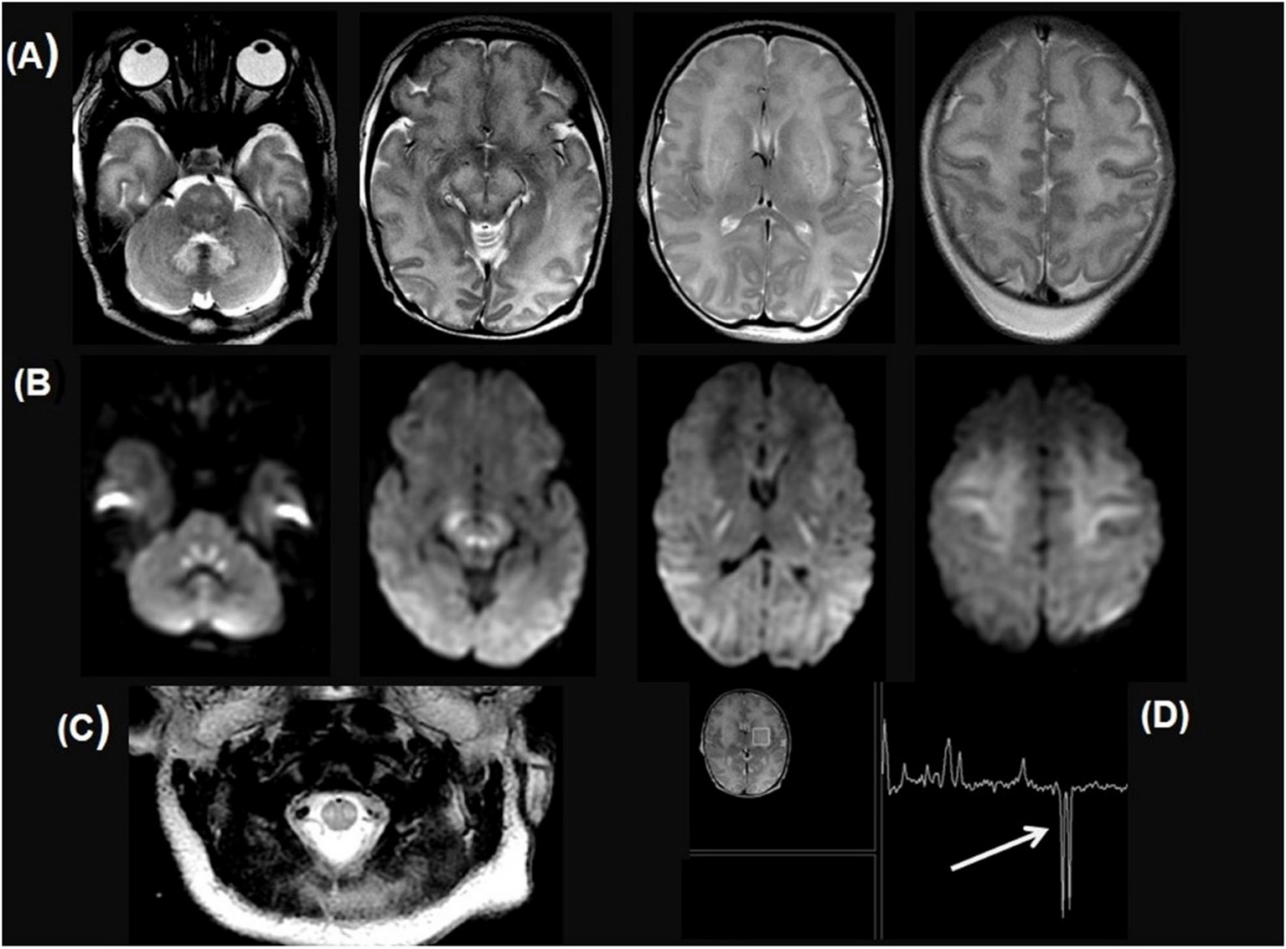
Ten-day-old newborn with *NDUFS6* variant. Axial T2W brain MRI images **(A)**, DWI images **(B)**, and H-MR spectroscopy with TE 144 **(C)** show T2 hyperintensity of dentate nuclei, pontine tegmentum and corticospinal tract, dorsal mesencephalon and contiguous cerebral peduncles, internal capsules, basal ganglia, medial thalami, and supratentorial white matter, with symmetric restricted diffusion in the cerebellar peduncles, central tegmental and corticospinal tracts, perirolandic subcortical and deep white matter. **(C)** Axial T2W image of the cervical cord shows symmetrical abnormal dorso-lateral bright T2 signal including likely the corticospinal tract. **(D)** MR spectroscopy shows markedly elevated Lactate at 1.33 ppm (arrows).

**Figure 3:**
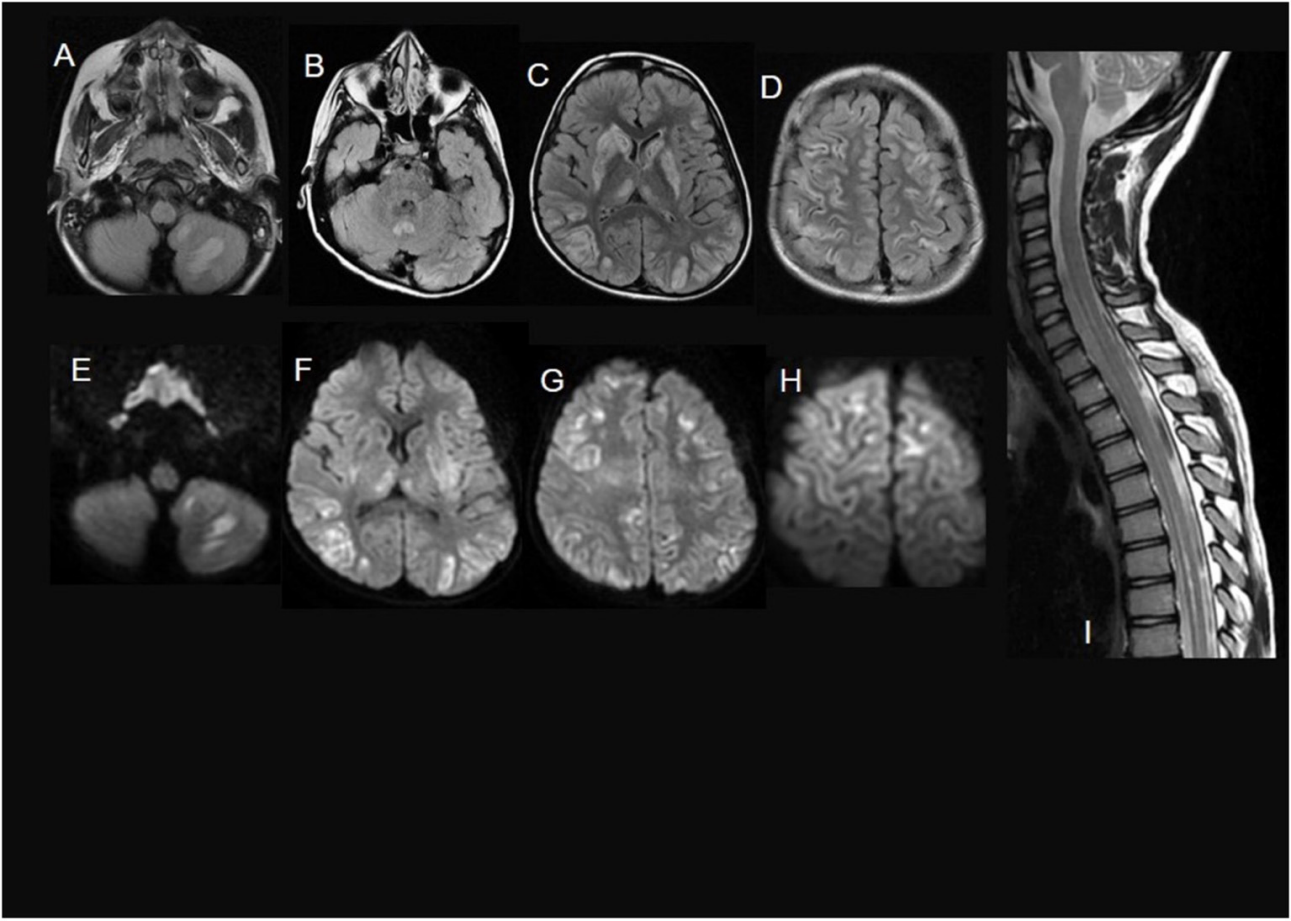
A 7-year-old child with Biotin thiamine responsive basal ganglia disease due to *SLC19A3* mutation. **(A–D)** axial FLAIR sections show multiple bilateral infra and supratentorial FLAIR hyperintense lesions involving the striatum, the medial thalami, the vermis, and the cortical/juxtacortical fronto-parietal and temporal areas. In **(E–H)**, diffusion restriction in many of these lesions is noted. Sagittal T2WI **(I)** shows an intramedullary T2 hyperintense lesion along with the lower cervical and dorsal cord till level opposite to 10th thoracic vertebra.

### Alexander Disease

Alexander disease, caused by *GFAP* variants, is characterized by defects in the synthesis or maintenance of the myelin sheath (12). The clinical manifestations of early-onset Alexander disease include megalencephaly, difficulty in swallowing, cognitive defects, seizures, and spasticity. MRI findings typically show bilateral white-matter lesions with frontal predominance. Patients with late-onset Alexander disease typically present with brainstem-spinal cord junction dysfunction and rarely have seizures or megalencephaly. Late-onset Alexander disease imaging demonstrates more involvement of the hindbrain structures, including the cerebellum, brainstem, and cervical spinal cord, and less involvement of the forebrain. Periventricular hyperintensities are also described. The hindbrain lesions may evolve to so-called tadpole-like atrophy of the medulla oblongata and cervical spinal cord (13).

### Krabbe Disease

Krabbe disease is an autosomal recessive disorder caused by a deficiency in galactocerebrosidase (galactosylceramide beta-galactosidase) activity. It has been classified into early-onset (<6 months) and late-onset (>6 months) variants. Clinical features of early-onset disease include severe progressive cognitive and motor deficits, visual loss, and seizures leading to death, usually by the age of 2 years. The typical presentation in late-onset disease is psychomotor regression, spastic paraparesis, and visual problems. Spinal MRI abnormalities include corticospinal tract involvement, posterior predominance, cauda equina enhancement, or atrophy of the cervical spinal cord (14).

## Mitochondrial Diseases

Mitochondrial diseases are clinically heterogeneous disorders caused by a wide spectrum of mutations in genes encoded mostly by the nuclear or mitochondrial genome in small fractions (2). These diseases usually affect multiple organs, frequently involving the central nervous system. Mitochondrial diseases mainly involve the brain, but the spinal cord is an increasingly recognized site of involvement. Spinal cord involvement in mitochondrial diseases most frequently occurs with brain involvement, but it may also be the only disease manifestation (4, 5). Early recognition of spinal cord involvement in mitochondrial diseases is essential because it may influence management. The list of mitochondrial diseases with spinal cord involvement is expanding (Table 1) (21–31) and is frequently reported in ISCA2-related multiple mitochondrial dysfunction syndrome 4 (24); Leigh syndrome; leukoencephalopathy with the brain stem, spinal cord involvement, and lactate elevation (10); myoclonic epilepsy with ragged-red fibers (22);

Kearns-Sayre syndrome (23); infantile-onset spinocerebellar ataxia; mitochondrial recessive ataxia syndrome (25); mitochondrial multiorgan dysfunction syndrome (4), more rarely in mitochondrial encephalopathy, lactic acidosis, and stroke-like episodes (MELAS) (21); chronic progressive external ophthalmoplegia (28); KARS-related disease (29); and Leber's hereditary optic neuropathy (30). Spinal cord involvement presents as weakness, sensory loss, autonomic disturbances, and bowel/bladder dysfunction. These abnormalities may be the initial manifestations of mitochondrial diseases, such as in Leigh syndrome, or may overlap with seizures, ataxia, headache, or movement disorders in cases of brain involvement (4, 5). With the progression of mitochondrial diseases, spinal cord involvement may advance as well.

**Figure 4:**
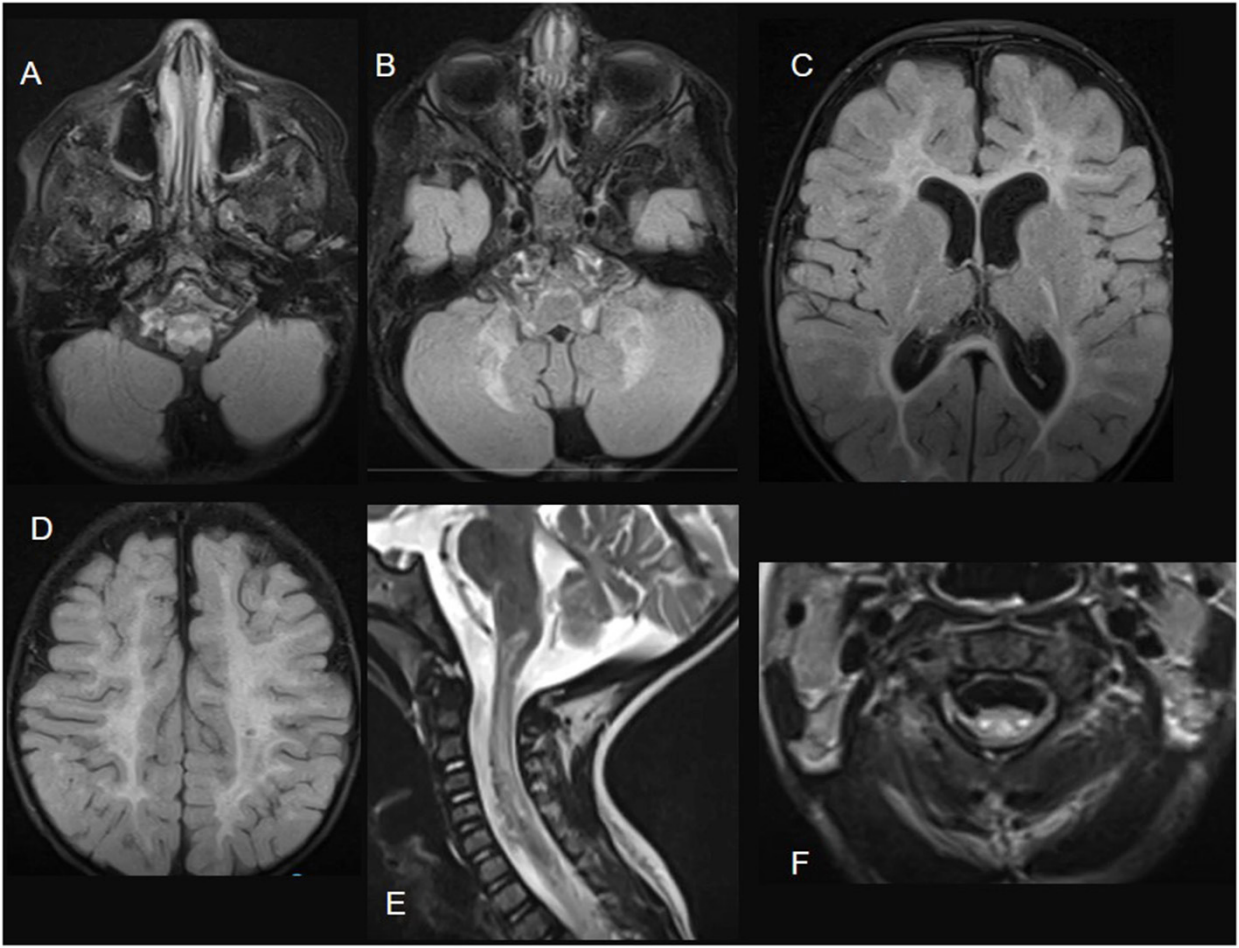
*ISCA2*-related disease. Brain MRI images; **(A–D)** axial FLAIR sections showing extensive diffuse bilateral supra and infratentorial signal abnormalities involving subcortical, deep, and subcortical white matter. Sagittal **(E)** and axial **(F)** T2-weighted images demonstrate spinal cord white matter involvement of the dorsal columns and the lateral corticospinal tracts.

Based on imaging or pathological findings, the most frequently involved regions in the spinal cord in mitochondrial diseases are the dorsal columns, corticospinal tracts, and spinocerebellar tracts. In *ISCA2*-related mitochondrial disease, imaging findings show extensive longitudinal involvement of the spinal cord, particularly the lateral corticospinal tract and dorsal column (Figure 4) (24). In MELAS, neuronal loss of the anterior and posterior horns and degeneration of the corticospinal tracts are observed (21). In *KARS*-related mitochondrial disease, it was described as “track-like” calcifications along the entire spinal cord (29).

## Deficiency-Related Metabolic Diseases

### Cobalamin-Related Remethylation Disorders cblC, cblD, cblE, cblF, cblG, cblJ, and MTHFR Deficiency

Genetic defects that affect the remethylation pathway cause hyperhomocysteinemia (32, 33). The clinical presentation of remethylation disorders varies; however, it mainly includes neurological manifestations (seizures, movement disorders, cognitive/behavior disorders, and microcephaly) and hematological features (megaloblastic anemia). Myelopathy is also a common feature of remethylation disorders and can be the presenting symptom in late-onset or untreated patients. Clinical manifestations include progressive abnormal gait, numbness, and sphincter dysfunction. Imaging changes always involve the posterior and lateral columns of the spinal cord, with the neuropathologic change showing vacuolation of the white matter, conceived as spongiform demyelination (32, 33). Early treatment of remethylation disorders is crucial and based on an appropriate diet and parenteral hydroxocobalamin, folate, and betaine administration in individuals with MTHFR deficiency.

### Biotinidase Deficiency

Biotinidase deficiency is an autosomal recessive disorder that manifests during the first few months of life with seizures, hypotonia, hearing, visual loss, skin rashes, and alopecia. Spinal cord involvement is usually a manifestation of late-onset biotinidase deficiency. Clinically, patients with biotinidase deficiency exhibit limb weakness (spastic paraplegia/tetraplegia) with or without vision problems. Imaging findings show long-segment spinal cord involvement (34). Postmortem studies have shown loss of myelination and necrotizing lesions in the deep cerebral gray matter, brainstem, and spinal cord. The diagnosis of biotinidase deficiency is established by analyzing serum levels of the biotinidase enzyme and the *BTD* gene. Early treatment with biotin (10–20 mg/day) markedly improves or completely resolves the myelopathy in biotinidase deficiency, but if the treatment is delayed, the symptoms may be irreversible.

### Ataxia With Vitamin E Deficiency

This rare autosomal recessive disease is caused by a mutation in the alpha-tocopherol transfer protein gene. Pathological studies of postmortem examinations have shown neuronal atrophy, axonal spheroids with a predilection for dorsal columns, and sensory demyelination. Neurologic phenotypes vary from progressive cerebellar ataxia and lower limb areflexia in early-onset disease to subacute combined degeneration with more pronounced peripheral neuropathy in late-onset disease. Spinal imaging findings are non-specific and may show changes in the posterior columns, particularly in the lower cervical segments. These abnormalities are associated with cerebellar atrophy in half of the affected cases (36). Early treatment with high-dose vitamin E supplementation has resulted in good outcomes in the treatment of ataxia with vitamin E deficiency, commonly seen in the Mediterranean region.

## Discussion

Spinal cord involvement, either clinical and/or radiological, in pediatric-onset metabolic disorders with Mendelian and mitochondrial inheritance is likely more frequent than anticipated and requires special attention. In inherited metabolic diseases, spinal cord involvement is most frequently reported in adrenoleukodystrophy, biotinidase deficiency, arginase deficiency, and cobalamin-related remethylation disorders cblC, cblD, cblE, cblF, cblG, cblJ, and MTHFR deficiency (9, 32–34, 41). In mitochondrial disorders, spinal cord involvement is most frequently reported in leukoencephalopathy with brainstem, spinal cord involvement and lactate elevation, Leigh syndrome, and multiple mitochondrial dysfunction syndrome (4). Spinal cord involvement in pediatric-onset metabolic disorders with Mendelian and mitochondrial inheritance must be considered a differential diagnosis of abnormalities more often found in acquired spinal cord diseases such as inflammation, demyelination, infection, or neoplastic, vascular, or toxic diseases (3–6).

The combination of careful clinical evaluation, brain and spine MRI, and metabolic/genetic investigations will help assess the frequency and pattern of spinal cord involvement in various inherited metabolic and mitochondrial myelopathies. Clinical manifestations of spinal cord involvement include weakness, sensory and autonomic disturbances, and sphincter dysfunction. These abnormalities may overlap with cerebral manifestations in cases with cerebral involvement. In some cases, an affected spinal cord may be the initial manifestation, such as in Leigh syndrome. Compared to acquired spinal cord diseases, heritable diseases in patients who exhibit myelopathic signs are more likely to have an insidious onset, progressive disease course, involvement of other systems (such as the eye, heart, skin, and kidneys), and a positive family history of a related disease. Several “red flag” MRI characteristics should prompt careful consideration of specific genetic metabolic or mitochondrial disorders. Although it is true that there is no uniform pattern of spinal cord involvement in metabolic disorders with Mendelian and mitochondrial inheritance, the dorsal columns, corticospinal tracts, or spinocerebellar tracts are most frequently involved, and gadolinium enhancement and significant spinal cord atrophy are absent (4, 7). However, some important exceptions to these general rules need to be mentioned: a selective spinal signal change in the dorsal columns could be found in patients with acquired dorsal root ganglion alteration (i.e., Sjogren syndrome), and gadolinium enhancement has been reported in some genetic (DARS mutation, Alexander disease, biotinidase deficiency) and acquired (B12 deficiency and methotrexate toxicity) conditions, which can mimic inflammatory conditions (8).

Advanced metabolic and genetic investigations to rule out metabolic disorders with Mendelian and mitochondrial inheritance are mainly based on the clinical phenotype and neuroimaging findings. In cases of high suspicion, targeted biomarker analysis or single gene analysis may facilitate the diagnostic process of treatable disorders and the initiation of disease-specific treatment. If the clinical and neuroimaging findings are non-specific and no specific cause is suspected, next-generation sequencing is a useful tool. In recent years, advanced genetic testing techniques have become efficient strategies to diagnose rare disease-causing mutations in children with metabolic disorders with Mendelian and mitochondrial inheritance. Timely diagnosis and management of such conditions is essential because early identification and treatment may limit the morbidity and mortality associated with metabolic disorders with Mendelian and mitochondrial inheritance. It is also important to anticipate the natural history of the newly diagnosed disorder and provide accurate genetic counseling.

## Ethics Statement

Written informed consent was obtained from the parent of the participant for the publication of any potentially identifiable images or data included in this article.

## Author Contributions

All authors participated in gathering the data, designing the article, and discussing and editing the manuscript.

## Conflict of Interest

The authors declare that the research was conducted in the absence of any commercial or financial relationships that could be construed as a potential conflict of interest.
